# Selective Electrical Tuning of Triple-Mode Strong Exciton–Plasmon Coupling in a WS_2_/J-Aggregates/Au@Ag Heterocavity

**DOI:** 10.3390/nano16120758

**Published:** 2026-06-16

**Authors:** Yufeng Hu, Zhiyuan Li, Qinglong Peng, Chen Xu, Yinyin Jiao, Lan Jiang, Kun Liang

**Affiliations:** 1 State Key Laboratory of Information Photonics and Optical Communications, School of Physical Science and Technology, Beijing University of Posts and Telecommunications, Beijing 100876, China; huyufeng@bupt.edu.cn (Y.H.); 2024111501@bupt.cn (Z.L.); 2024111502@bupt.cn (Q.P.); 2023212517@bupt.cn (C.X.); 2022jianglan@bupt.edu.cn (L.J.); 2 School of Information and Communication Engineering, Beijing University of Posts and Telecommunications, Beijing 100876, China; jyy19996289849@bupt.edu.cn

**Keywords:** strong coupling, electrically tunable polaritons, plasmonic nanocavity, transition metal dichalcogenides, polaritonics

## Abstract

Active control of multi-mode light–matter interactions is crucial for advancing quantum photonic technologies. Although triple-mode plasmon–exciton systems involving two distinct excitonic transitions offer a pathway to multi-level polaritonic states, achieving reversible electrical tuning at room temperature remains challenging. Here, we numerically investigate an electrically tunable triple-mode strong-coupling system comprising a J-aggregate-coated Au@Ag nanorod coupled with monolayer WS_2_. The simulated spectra show a UPB–LPB energy separation of approximately 239 meV near the zero-detuning condition. A modest gate voltage (2.0 V to 3.8 V) selectively modulates the middle and lower polariton branches over ∼46 meV, while the upper branch remains largely unaffected. This selective control is elucidated via a triple-mode coupled-oscillator model and Hopfield coefficient analysis, linking the polariton response to the excitonic composition. These results establish a framework for electrically reconfigurable multi-level polaritonic devices, offering potential for ultracompact optical modulators, high-sensitivity multiplexed sensors, and programmable quantum photonic circuits.

## 1. Introduction

Manipulating light–matter interactions at the nanoscale is essential for the advancement of integrated photonic and quantum optical technologies [1,2,3,4]. Such control underpins key applications, including on-chip optical communication [5,6], scalable quantum networks [7,8], and ultrasensitive sensing [9,10,11]. When the coherent exchange rate between a photonic mode and an electronic transition exceeds their respective dissipation rates, the system enters the strong coupling regime, forming exciton–polaritons with hybrid photonic–excitonic character [12,13,14,15]. These part-light, part-matter polaritons combine optical and electronic properties, enabling novel functionalities such as room-temperature polariton lasing [16,17,18], ultrafast optical switching [19], and robust quantum information processing [20,21,22].

Early studies of strong coupling primarily focused on two-mode systems, in which a single type of quantum emitter interacts with one cavity mode to form two polaritonic states [23,24]. However, emerging applications in quantum networks, optical information processing, and reconfigurable nanophotonics increasingly require multi-mode polaritonic systems capable of constructing more complex hybrid energy landscapes [25,26]. In this context, triple-mode exciton–plasmon coupling in plasmonic nanocavities has attracted growing attention, because one plasmonic mode can simultaneously mediate interactions with two distinct excitonic transitions [27,28]. Hybrid organic–inorganic heterostructures that combine Frenkel excitons from J-aggregates with Wannier–Mott excitons in monolayer transition-metal dichalcogenides (TMDCs) are particularly appealing. Such systems integrate the high oscillator strength and narrow linewidths of organic aggregates with the strong Coulomb interaction, large exciton binding energy, and electrical tunability of two-dimensional semiconductors [29,30,31,32,33].

Recent breakthroughs have demonstrated room-temperature triple-mode strong coupling in hybrid plasmonic systems [34]. In particular, platforms combining gold nanostructures with J-aggregates and TMDCs such as WS_2_ have shown three distinct polariton branches, confirming simultaneous coupling between a plasmon resonance and two different excitonic transitions [35,36]. Despite these advances, the tuning of multi-mode polariton states has largely relied on thermal modulation, passive environmental changes, or geometry-dependent fabrication, which limits the speed, reversibility, and device-level scalability of such systems [32]. Active electrical control offers a more practical route toward dynamically reconfigurable polaritonic devices. A pioneering demonstration was reported in a WS_2_/organic dye microcavity, where the hybrid polariton composition was modulated by altering the exciton–trion balance with an in-plane electric field [37]. Nevertheless, a comprehensive theoretical framework for designing, optimizing, and electrically reconfiguring triple-mode exciton–plasmon coupling in nanoscale plasmonic cavities remains lacking. Key challenges include optimizing nanocavity geometry for maximal tunability, quantifying voltage-dependent energy redistribution among the plasmonic and excitonic components, and identifying device functionalities enabled by branch-selective electrical control.

In this work, we present a numerical investigation of a room-temperature triple-mode strong-coupling platform based on a WS_2_/J-aggregate/Au@Ag hybrid nanocavity. The analysis is entirely based on full-wave finite-difference time-domain simulations combined with a triple-mode coupled-oscillator model. Leveraging the enhanced field confinement and spectral tunability provided by the Au@Ag nanocavity, a localized plasmonic resonance hybridizes simultaneously with two excitonic species: Frenkel excitons from J-aggregates and Wannier–Mott excitons in monolayer WS_2_, yielding three distinct polariton branches with UPB-LPB energy separation up to ∼239meV near the zero-detuning condition. To enable electrically programmable polariton engineering, we incorporate the gate-dependent dielectric response of WS_2_ into a triple-mode coupled-oscillator framework combined with full-wave electrodynamic simulations. We systematically map how nanocavity geometry, applied gate bias, and background refractive index govern the hybrid-mode energies and compositions. Importantly, we reveal branch-selective electrical programmability: the middle and lower polariton branches can be tuned over a large range (up to ΔE≈46meV), whereas the upper branch remains nearly unchanged over the same bias range, consistent with its minimal WS_2_ fraction extracted from Hopfield-coefficient analysis. These results provide routes for designing electrically reconfigurable polaritonic platforms, with potential applications in ultracompact optical modulator, multiplexed sensing, and programmable quantum photonic devices.

## 2. Results and Discussion

**Figure 1:**
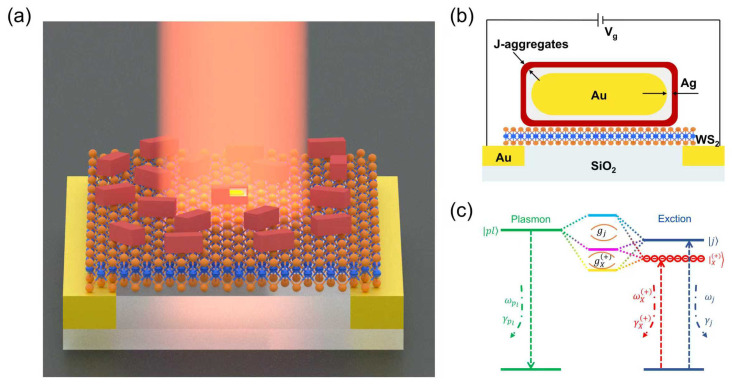
Conceptual framework for the electrically tunable triple-mode strong coupling system. (**a**) Schematic illustration of the hybrid plexcitonic system. (**b**) Cross-sectional view of the device architecture illustrating the electrical gating scheme. (**c**) Energy level diagram of the triple-mode strong coupling interaction.

The conceptual framework of the electrically tunable triple-mode strong coupling system is illustrated in Figure 1. The core of the system is a hybrid plexcitonic heterostructure, schematically shown in Figure 1a. It is composed of three components: (i) an Au@Ag core–shell nanorod supporting a localized plasmonic mode with resonance energy $\omega_{pl}$, (ii) a layer of J-aggregates providing a stable, high-oscillator-strength Frenkel exciton $\omega_{j}$, and (iii) a monolayer of WS_2_ hosting Wannier–Mott excitons at $\omega_{X}$. Applying a gate voltage across the WS_2_ layer modulates its carrier density (Figure 1b), which alters the optical properties of WS_2_, primarily its exciton energy and oscillator strength. The underlying physical mechanism is depicted in the energy-level diagram (Figure 1c), where the plasmon mode acts as a bridge simultaneously coupling to the two energetically detuned excitonic species. When the coupling is sufficiently strong ($g_{j}>\frac{\gamma_{pl}+\gamma_{j}}{2}$, $g_{x}>\frac{\gamma_{pl}+\gamma_{x}}{2}$), the initial uncoupled states hybridize, leading to the formation of three new polaritonic eigenstates. A crucial feature of our system is the electrically tunable WS_2_-plasmon coupling strength $g_{X}^{\left(+\right)}$, which provides a direct route to actively reconfigure the entire coupled energy landscape.

**Figure 2:**
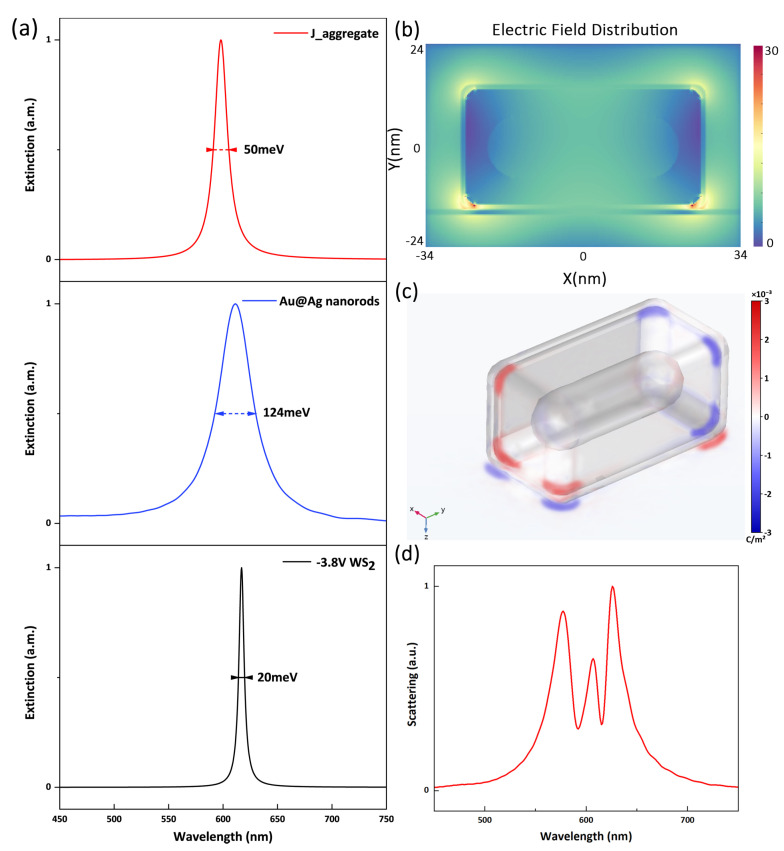
Spectral characteristics and near-field properties of the triple-mode plexcitonic system. (**a**) Calculated extinction spectra of the three uncoupled components: the Au@Ag nanorod with a broad plasmon resonance centered at 592 nm, the J-aggregate layer with a Frenkel exciton absorption peak at 599 nm, and monolayer WS_2_ with a Wannier–Mott exciton resonance at 617 nm. The corresponding FWHM values are ∼124 meV, ∼50 meV, and ∼20 meV, respectively. (**b**) Simulated electric-field intensity distribution, ${\left|E\right|}^{2}/{\left|E_{0}\right|}^{2}$, of the Au@Ag nanorod at the plasmon resonance, showing pronounced field confinement at the nanorod corners. (**c**) Corresponding surface charge distribution of the Au@Ag nanorod, where the opposite charge accumulation at the two ends confirms the excitation of a longitudinal dipolar plasmon mode. (**d**) Representative scattering spectrum of the coupled WS_2_/J-aggregate/Au@Ag heterostructure under near-resonant conditions, exhibiting three well-resolved polaritonic resonances assigned to the upper (UPB), middle (MPB), and lower (LPB) polariton branches.

To establish the physical basis for electrically tunable triple-mode strong coupling, we first examine the spectral properties of the individual components and the near-field response of the plasmonic nanocavity. The excitonic resonance energies and linewidths of the J-aggregate and monolayer WS_2_ were parameterized using the reported experimental spectra in Refs. [36] and [31], respectively. The Au@Ag plasmon resonance and linewidth were extracted from the FDTD spectrum calculated using the experimentally tabulated optical constants and the geometry specified in Appendix A.1. Figure 2a shows the calculated extinction spectra of the three uncoupled constituents. The Au@Ag core–shell nanorod exhibits a broad plasmon resonance centered at 592 nm, with a full width at half maximum (FWHM) of 124 meV. In contrast, the J-aggregate layer shows a much narrower Frenkel exciton absorption peak at 599 nm, with an FWHM of 50 meV. The monolayer WS_2_ presents a sharp Wannier–Mott exciton resonance at 617 nm, with an FWHM of 20 meV. The near-resonant spectral alignment of these three modes, together with their distinct linewidth hierarchy, provides favorable conditions for multi-mode hybridization.

The strong light–matter interaction in this system is further supported by the near-field characteristics of the Au@Ag nanocavity. As shown in Figure 2b, the simulated cross-sectional distribution of ${\left|E\right|}^{2}/{\left|E_{0}\right|}^{2}$ near the plasmon resonance reveals pronounced field confinement at the nanorod corners, forming nanoscale hotspots that enhance the local electromagnetic density and facilitate exciton–plasmon coupling. The corresponding surface charge distribution in Figure 2c confirms that this field enhancement originates from a longitudinal dipolar plasmon mode, as indicated by the accumulation of positive and negative charges at opposite ends of the nanorod. When the Au@Ag nanorod, J-aggregates, and monolayer WS_2_ are integrated into a hybrid heterostructure, the single broad plasmonic resonance evolves into three well-resolved polaritonic resonances in the scattering spectrum (Figure 2d). These resonances can be assigned to the upper, middle, and lower polariton branches (UPB, MPB, and LPB), providing a clear spectral signature of triple-mode exciton–plasmon coupling. Notably, the emergence of the MPB distinguishes this system from conventional two-mode strong coupling platforms.

**Figure 3:**
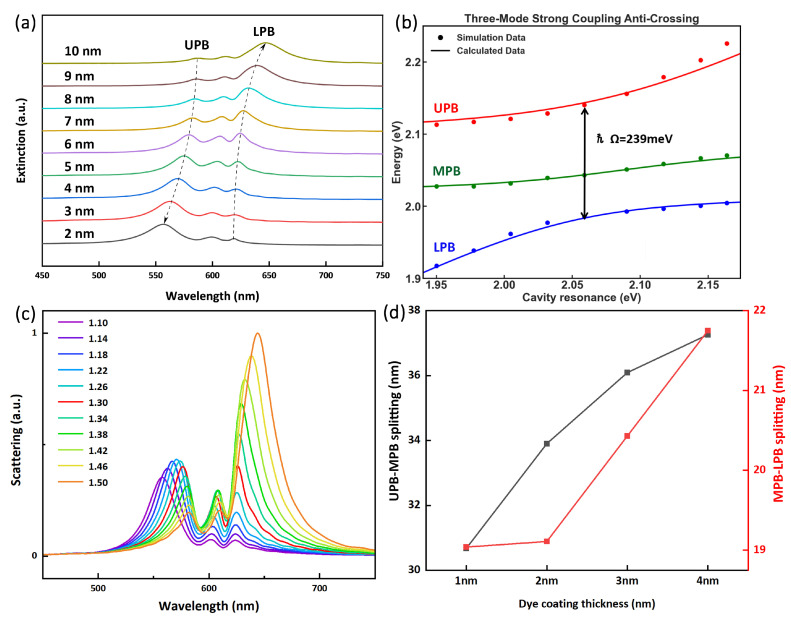
Multiparameter tuning of the triple-mode plexcitonic system. (**a**) Calculated extinction spectra of the coupled WS_2_/J-aggregate/Au@Ag heterostructure under a Ag shell-thickness sweep of $t_{\mathrm{Ag}}=2$–10 nm, corresponding to an aspect ratio variation from 1.73 to 2.45. (**b**) Anticrossing analysis based on a triple-mode coupled-oscillator model. The symbols represent the simulated peak positions, the solid curves denote the fitted polariton branches, and the dashed lines indicate the uncoupled plasmon, J-aggregate exciton, and WS_2_ exciton energies. A large UPB-LPB energy separation of $\Delta E_{\mathrm{UPB}-\mathrm{LPB}}=239\;\mathrm{meV}$ is obtained near the zero-detuning condition. (**c**) Calculated scattering spectra of the coupled heterostructure as the environmental refractive index is varied from n=1.1 to 1.5. (**d**) Spectral separations between adjacent polariton branches as a function of J-aggregate coating thickness, revealing enhanced collective coupling with increasing Frenkel exciton density.

To evaluate the tunability of the triple-mode strong coupling, we first investigate how the nanocavity geometry modulates the plexcitonic states. As shown in Figure 3a, the Ag-shell thickness is varied from 2 to 10 nm, corresponding to an increase in the aspect ratio (AR) of the Au@Ag nanorod from 1.73 to 2.45. Increasing the AR redshifts the bare plasmon resonance, thereby sweeping the detuning between the plasmon mode and the two excitonic transitions. Here, the detuning is defined as $\Delta =E_{pl}-\frac{1}{2}\left(E_{J}+E_{X}\right)$, where $E_{pl}$, $E_{J}$, and $E_{X}$ denote the plasmon, J-aggregate exciton, and WS_2_ exciton energies, respectively. By extracting the spectral peak positions from the simulated extinction spectra in Figure 3a, we construct the anticrossing dispersion diagram shown in Figure 3b. The upper, middle, and lower polariton branches (UPB, MPB, and LPB) clearly deviate from the uncoupled plasmon and exciton energies, exhibiting the characteristic anticrossing behavior of triple-mode strong coupling. Fitting the dispersion with a triple-mode coupled-oscillator Hamiltonian yields a large mode splitting of ℏΩ≈239meV near the zero-detuning condition, further confirming the strong hybridization among the plasmon mode, J-aggregate exciton, and WS_2_ exciton.

Next, we examine the response of the triple-mode plexcitonic system to changes in the surrounding dielectric environment, which is important for refractive index sensing applications. As shown in Figure 3c, when the environmental refractive index is increased from n=1.10 to 1.50, all three polaritonic resonances exhibit a systematic redshift. This collective spectral shift arises because each polariton branch retains a finite plasmonic component and therefore inherits the strong environmental sensitivity of the Au@Ag plasmon mode. The simultaneous shift of the UPB, MPB, and LPB provides multiple spectral channels for refractive-index-based sensing. We further evaluate how the coupling can be engineered by controlling the effective number of excitons participating in the interaction. Figure 3d shows the dependence of the spectral separation between adjacent polariton branches on the J-aggregate coating thickness. As the dye layer thickness increases from 1 to 4 nm, more Frenkel excitons overlap with the nanocavity hotspots, leading to enhanced collective exciton–plasmon coupling. This enhancement is reflected by the monotonic increase in the UPB-MPB and MPB-LPB separations.

After examining passive geometric and environmental tuning, we next focus on active electrical modulation of the triple-mode strong coupling system. Applying a gate voltage across the Au@Ag nanorod/WS_2_ heterostructure generates a vertical electric field in the nanogap, allowing the excitonic response of monolayer WS_2_ to be dynamically controlled. This field-induced modulation provides an effective route to reconfigure light–matter interactions in the hybrid nanocavity.

In the present gated heterostructure, the local electric field experienced by monolayer WS_2_ is affected by the dielectric stack, electrode geometry, voltage drop, carrier screening, and nanocavity-induced field redistribution. Therefore, we do not assume a strictly uniform microscopic field across WS_2_ or use a simple relation such as $F=V_{g}/d$. Instead, the gate-induced excitonic response is incorporated through an effective voltage-dependent dielectric model calibrated from experimentally reported WS_2_ spectra. Formally, the effective field can be written as

(1) $$F_{\mathrm{eff}}\left(V_{g}\right)=\eta \frac{V_{g}-V_{0}}{d_{\mathrm{eff}}},$$

where $V_{0}$ is the reference gate voltage, $d_{\mathrm{eff}}$ denotes the effective electrostatic thickness, and η accounts for voltage-drop and screening effects. In the simulations, the voltage-dependent exciton resonance energy, linewidth, and oscillator strength are used directly as calibrated effective parameters. To describe this process, we formulate an analytical expression for the complex dielectric function of monolayer WS_2_ under an external vertical electric field $F_{\mathrm{eff}}\left(V_{g}\right)$ based on quantum perturbation theory. The model captures the electric-field dependence of both the exciton resonance energy and the oscillator strength. Specifically, the exciton energy shift is described by the linear Stark effect, while the reduction in oscillator strength arises from field-induced electron–hole separation and exciton wavefunction delocalization. By incorporating these two effects, the model provides a theoretical basis for predicting the gate-dependent optical response of WS_2_ and for designing electrically tunable exciton–plasmon coupling devices.

The gate-induced exciton shift is described as an effective voltage-dependent Stark-like response. In a system possessing inversion symmetry or out-of-plane mirror symmetry, the first-order Stark correction vanishes in the absence of a permanent excitonic dipole. In the present gated heterostructure, the asymmetric dielectric and electrode environment, together with dielectric screening and nanocavity-induced field redistribution, breaks the out-of-plane symmetry and can permit an effective first-order contribution over the limited voltage range considered here. Because the microscopic asymmetry and local electric field cannot be uniquely determined without a full electrostatic model, the exciton energy is parameterized directly in terms of the applied gate voltage:

(2) $$E_{X}\left(V_{g}\right)=E_{X}\left(V_{0}\right)-\beta \left(V_{g}-V_{0}\right),$$

Here, $V_{0}$ is the reference gate voltage and β is an experimentally calibrated effective voltage-dependent coefficient. It should not be interpreted as a universal intrinsic Stark coefficient of monolayer WS_2_, but rather as a device-dependent parameter incorporating the effects of voltage drop, dielectric screening, structural asymmetry, and local field redistribution.

The second field-induced mechanism is the reduction of the exciton oscillator strength caused by electron–hole wavefunction delocalization. Under a vertical electric field, the electron and hole wavefunctions are pulled in opposite directions, which reduces their spatial overlap. Since the exciton oscillator strength is proportional to the squared electron–hole overlap integral, it can be expressed as

(3) $$f_{i}\left(F\right)\propto {\left|\langle \psi_{e}\left(F\right)|\psi_{h}\left(F\right)\rangle \right|}^{2}$$

Within an effective variational picture, the field-induced expansion and separation of the exciton wavefunction can be described by a field-dependent length scale β(F). As *F* increases, β(F) increases, indicating a reduced electron–hole overlap. For moderate field strengths, this overlap reduction can be approximated by an exponential dependence on $F^{2}$:

(4) $${\left|\langle \psi_{e}\left(F\right)|\psi_{h}\left(F\right)\rangle \right|}^{2}\approx {\left|\langle \psi_{e}\left(0\right)|\psi_{h}\left(0\right)\rangle \right|}^{2}\exp\left(-\frac{F^{2}}{F_{0}^{2}}\right)$$

where $F_{0}$ is a characteristic electric field that describes the onset of significant field-induced exciton delocalization. Accordingly, the oscillator strength is modeled as

(5) $$f_{i}\left(F\right)=f_{i}\left(0\right)\exp\left(-\frac{F^{2}}{F_{0}^{2}}\right)$$

This phenomenological form captures the essential physical effect that a stronger vertical electric field weakens the excitonic transition by reducing the electron-hole overlap.

The characteristic electric field $F_{0}$ is defined as $F_{0}=\frac{E_{b}}{ea_{X}}$, where $E_{b}$ is the exciton binding energy and $a_{X}$ is the exciton Bohr radius. Physically, $F_{0}$ represents the field scale at which the applied electric field becomes strong enough to substantially perturb the bound electron–hole pair. Combining the linear Stark shift of the exciton resonance with the field-induced reduction of oscillator strength, the field-dependent exciton energy and oscillator-strength parameter can be written as $E_{i}\left(F\right)=E_{i}\left(0\right)-\alpha F$, and $f_{i}\left(F\right)=f_{i}\left(0\right)\exp\left(-\frac{F^{2}}{F_{0}^{2}}\right)$, respectively.

Using these two field-dependent quantities, the complex dielectric function of monolayer WS_2_ under a vertical electric field is modeled by a Lorentz-type expression:

(6) $$\varepsilon_{{\mathrm{WS}}_{2}}\left(E,F\right)=\varepsilon_{\infty }+\frac{f_{i}\left(0\right)\exp\left(-\frac{F^{2}}{F_{0}^{2}}\right)}{{\left[E_{i}\left(0\right)-\alpha F\right]}^{2}-E^{2}-i\Gamma_{i}E}$$

where E=ℏω, $\varepsilon_{\infty }$ is the high-frequency dielectric background, and $\Gamma_{i}$ denotes the exciton linewidth. This model simultaneously accounts for the electric field-induced shift of the exciton resonance and the suppression of its oscillator strength. It therefore provides the theoretical input for calculating the gate-dependent optical response of monolayer WS_2_ and its electrically tunable coupling with the Au@Ag plasmonic nanocavity.

**Figure 4:**
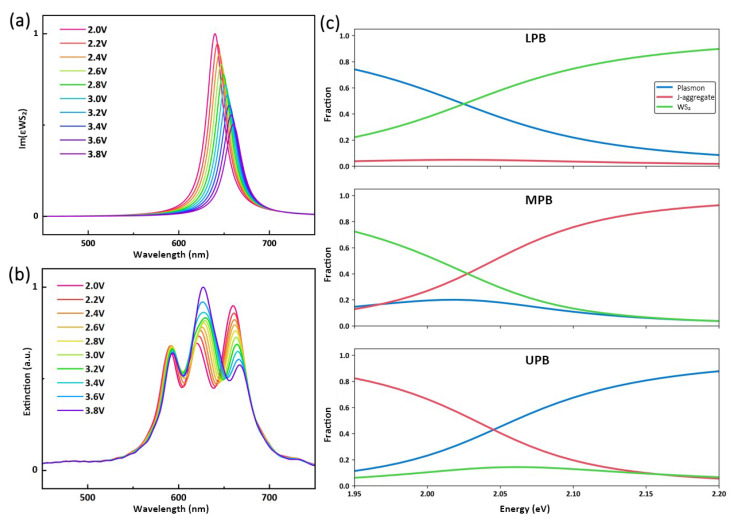
Electrical manipulation of the triple-mode strong coupling system. (**a**) Calculated imaginary part of the WS_2_ dielectric function under different gate voltages. (**b**) Extinction spectra of the coupled WS_2_/J-aggregate/Au@Ag heterostructure under the same gate-voltage range, demonstrating electrically tunable triple-mode exciton–plasmon coupling. (**c**) Hopfield coefficient analysis obtained from the coupled-oscillator model, showing the plasmon, J-aggregate exciton, and WS_2_ exciton fractions of the LPB, MPB, and UPB as a function of cavity-mode energy. The different WS_2_ exciton fractions near the corresponding cavity energies explain the branch-dependent electrical response.

This gate-dependent dielectric model enables monolayer WS_2_ to serve as an active excitonic element in the hybrid nanocavity. The calculated imaginary part of the dielectric function, Im(εWS2), is shown in Figure 4a. The model is calibrated at a gate bias of 2.0 V against the experimentally reported optical response of electrically driven WS_2_ [31]. Experimentally, electron depletion under negative gate bias gives rise to a sharp neutral-exciton resonance (X^0^) near 617 nm, whereas electron accumulation under positive bias transfers the spectral weight toward a broader, longer-wavelength resonance associated with charged excitons (trions, X^−^). In the present simulations, the calibrated gate-dependent dielectric response within $V_{g}=2.0$–3.8 V is used for polariton tuning. This gate-induced redistribution of excitonic oscillator strength and resonance energy is captured by the dielectric model, providing a tunable optical input for polariton engineering.

Using this gate-dependent WS_2_ response, we then calculate the extinction spectra of the coupled WS_2_/J-aggregate/Au@Ag heterostructure under different gate biases (Figure 4b). A pronounced branch-selective response is observed. The upper polariton branch (UPB) remains nearly unchanged over the applied bias range, whereas the middle and lower polariton branches (MPB and LPB) exhibit clear voltage-dependent shifts in both spectral position and intensity. The extracted tuning rates reach approximately 0.5 nm/0.1 V for the MPB and 0.75 nm/0.1 V for the LPB, indicating a highly efficient electrical modulation of the hybrid energy landscape. After converting the peak wavelengths into photon energies, the maximum voltage-induced energy shift reaches approximately 46 meV for the LPB and 30 meV for the MPB within the applied gate-voltage range. This selective tunability provides a direct route toward electrically programmable multi-level polaritonic devices.

To quantitatively explain the branch-selective electrical response, we employ a triple-mode coupled-oscillator model (TCOM) [34]:

(7) $$\hat{H}=\left(\begin{array}{ccc} E_{pl}-i\frac{\gamma_{pl}}{2} & g_{J} & g_{X}\left(V\right)\\ g_{J} & E_{J}-i\frac{\gamma_{J}}{2} & 0\\ g_{X}\left(V\right) & 0 & E_{X}\left(V\right)-i\frac{\gamma_{X}\left(V\right)}{2} \end{array}\right)$$

Here, $E_{pl}$, $E_{J}$, and $E_{X}$ denote the resonance energies of the plasmon mode, J-aggregate exciton, and WS_2_ exciton, respectively, while $\gamma_{pl}$, $\gamma_{J}$, and $\gamma_{X}$ are their corresponding linewidths. The coupling strengths between the plasmon mode and the two excitonic transitions are represented by $g_{J}$ and $g_{X}$, respectively. The zero off-diagonal term between the two excitonic states indicates that direct coupling between the J-aggregate exciton and the WS_2_ exciton is neglected, and their interaction is mediated by the plasmonic mode. Importantly, the WS_2_-related parameters, including $E_{X}\left(V\right)$, $\gamma_{X}\left(V\right)$, and $g_{X}\left(V\right)$, are voltage-dependent because of the gate-modulated excitonic response described above. The complex eigenvalues of this Hamiltonian yield the polariton energies and linewidths, while the corresponding eigenvectors provide the Hopfield coefficients of each polariton branch. By fitting the anticrossing dispersion in Figure 3b, the model gives a large UPB-LPB normal-mode splitting of $\Delta E_{\mathrm{UPB}-\mathrm{LPB}}$ = 239 meV near the zero-detuning condition, supporting the strong hybridization among the plasmon mode, J-aggregate exciton, and WS_2_ exciton.

As shown in Tables S1 and S2, a quantitative strong-coupling analysis based on bare-mode linewidths, fitted coupling strengths, polariton linewidths, cooperativity, and branch resolvability is provided. The individual channel cooperativities are calculated as: $C_{i}=\frac{4g_{i}^{2}}{\gamma_{\mathrm{pl}}\gamma_{i}}$. This yields $C_{J}=2.42>1$ and $C_{X}=1.42>1$, supporting strong coupling in both excitonic channels. Following the previously reported criterion for a three-mode plasmon–exciton system [28,36,38], the linewidth of each polariton branch is calculated from its normalized modal composition: $\kappa_{i}={\left|c_{\mathrm{pl}}\right|}^{2}\gamma_{\mathrm{pl}}+{\left|c_{J}\right|}^{2}\gamma_{J}+{\left|c_{X}\right|}^{2}\gamma_{X}$. At the near-zero detuning condition, the resulting linewidths are $\kappa_{\mathrm{UPB}}$ = 75.0 meV, $\kappa_{\mathrm{MPB}}$ = 84.6 meV, and $\kappa_{\mathrm{LPB}}$ = 90.4 meV. The corresponding normalized linewidth weights are defined as $\alpha_{i}=\frac{\kappa_{i}}{\kappa_{\mathrm{UPB}}+\kappa_{\mathrm{MPB}}+\kappa_{\mathrm{LPB}}}$, giving $\alpha_{\mathrm{UPB}}$ = 0.3000, $\alpha_{\mathrm{MPB}}$ = 0.3384, and $\alpha_{\mathrm{LPB}}$ = 0.3616. The linewidth-weighted resolving threshold is therefore $\alpha_{\mathrm{UPB}}\kappa_{\mathrm{UPB}}+\alpha_{\mathrm{MPB}}\kappa_{\mathrm{MPB}}+\alpha_{\mathrm{LPB}}\kappa_{\mathrm{LPB}}$ = 83.82 meV. The fitted UPB–LPB energy separation at near-zero detuning is 239 meV, which is substantially larger than this threshold. Together with the two cooperativities exceeding unity and the clearly resolved anticrossing branches, these results provide quantitative evidence that the observed three-branch response originates from triple-mode strong coupling.

The physical origin of the selective electrical modulation is clarified by the Hopfield coefficient analysis shown in Figure 4c. The right eigenvectors of the non-Hermitian Hamiltonian were normalized according to $\sum_{j}|c_{j}{|}^{2}=|c_{\mathrm{pl}}{|}^{2}+|c_{X}{|}^{2}+{\left|c_{J}\right|}^{2}=1$, where *j* denotes the cavity plasmon, J-aggregate exciton, and WS_2_ exciton components, respectively. This constraint ensures that the plotted mixing fractions sum to unity for each polariton branch across the entire detuning range. Crucially, given the lossy, non-Hermitian nature of this tripartite network, these coefficients should not be interpreted as conservative quantum probabilities in the strict Hermitian sense. Rather, they serve as normalized modal weights to dynamically track the redistribution of plasmonic and excitonic characters under voltage tuning, providing a framework for evaluating branch-selective modulation and hybrid polariton linewidth evolution. The UPB is mainly composed of the plasmon mode and the J-aggregate exciton, with only a negligible WS_2_ exciton fraction (<5%). As a result, it is weakly affected by the gate-induced modulation of WS_2_. In contrast, the MPB and LPB contain substantial WS_2_ excitonic contributions. The LPB is predominantly a WS_2_–plasmon hybrid state, whereas the MPB contains mixed contributions from all three components. Therefore, voltage-induced changes in WS_2_ exciton energy, linewidth, and oscillator strength directly perturb the MPB and LPB, leading to the branch-selective tuning observed in the triple-mode coupled system.

## 3. Conclusions

In conclusion, we have numerically investigated an electrically tunable triple-mode strong coupling system based on an Au@Ag nanorod/J-aggregate/WS_2_ heterostructure using full-wave electromagnetic simulations and a triple-mode coupled-oscillator model. By incorporating the gate-dependent dielectric response of monolayer WS_2_, we established a model that accounts for both the linear Stark shift of the exciton resonance and the field-induced reduction of oscillator strength arising from exciton wavefunction delocalization. Under near-zero detuning conditions, the hybrid nanocavity exhibits three well-resolved polariton branches with UPB-LPB energy separation of Δ$E_{\mathrm{UPB}-\mathrm{LPB}}=239\;\mathrm{meV}$, supporting strong hybridization among the plasmon mode, J-aggregate exciton, and WS_2_ exciton. More importantly, the system enables branch-selective electrical modulation of the polariton states. Within the applied gate-voltage range of 2.0–3.8 V, the middle and lower polariton branches show pronounced voltage-dependent changes in their spectral positions and intensities, with tuning rates of approximately 0.5 nm/0.1 V for the MPB and 0.75 nm/0.1 V for the LPB. In contrast, the upper polariton branch remains nearly unchanged because of its negligible WS_2_ exciton fraction and dominant contribution from the electric field-insensitive J-aggregate exciton. The triple-mode coupled-oscillator model and Hopfield coefficient analysis reveal that this selective modulation originates from the different WS_2_ excitonic weights in the three polariton branches. These findings provide a theoretical framework for electrically reconfigurable multi-mode polaritonic systems and highlight the potential of hybrid organic–inorganic plasmonic nanocavities for ultracompact optical modulation, multiplexed sensing, and programmable quantum photonic integration.

## Data Availability

All data are available from the authors upon reasonable request.

## Supplementary Materials

The following supporting information can be downloaded at: https://www.mdpi.com/article/10.3390/nano16120758/s1, Figure S1: Comparison of the gate-voltage-dependent exciton peak wavelengths and full widths at half maximum (FWHM) extracted from the experimentally reported WS_2_ spectra with the corresponding values obtained from the present Lorentz-type dielectric model. The fitted peak wavelengths agree well with the experimental data, with a root-mean-square error of (0.53 nm); Table S1: The linewidth data of plasmons, J-aggregates, WS_2_, and polaritons; Table S2: Cooperativity analysis; Table S3: Material parameters used in the numerical simulations.

## Appendix A. Methods

### Appendix A.1. Geometric Model of the Hybrid Heterocavity

The geometry of the Au@Ag nanorod was designed according to experimentally established seed-mediated growth characteristics of core–shell noble-metal nanorods, where the plasmon resonance can be tuned by controlling the Ag-shell thickness and the nanorod aspect ratio. In the numerical model, the Au core was constructed as a cylinder with a length of 24 nm capped by two hemispherical ends with a radius of 6 nm, giving a total core length of 36 nm and a diameter of 12 nm. The Ag shell was defined with a radial thickness of 4 nm and an axial thickness of 5 nm at both longitudinal ends. To avoid unphysical field singularities at sharp metallic tips, the tip curvature radius of the Ag shell was set to 2 nm. The Au@Ag nanorod was then conformally coated with a 1 nm thick J-aggregate layer, resulting in a final coated nanorod with a total length of 48 nm and a diameter of 22 nm. The coated nanorod was placed in direct contact with a monolayer WS_2_, which was modeled with a thickness of 1 nm to represent the van der Waals heterostructure.

### Appendix A.2. FDTD Simulations

The optical response and near-field distribution of the hybrid heterocavity were calculated using the finite-difference time-domain (FDTD) method. Perfectly matched layer (PML) absorbing boundary conditions were applied in all three spatial directions to suppress artificial reflections from the simulation boundaries. A total-field scattered-field (TFSF) source was used to excite the structure and to separate the incident and scattered fields, enabling the extraction of the extinction and scattering spectra.

To accurately resolve the strongly confined electromagnetic fields in the nanocavity region, an override mesh with a spatial resolution of 0.1 nm was applied around the Au@Ag nanorod, J-aggregate coating, and monolayer WS_2_. This fine mesh was used to capture the nanoscale hotspots near the metallic nanorod and the sub-nanometer-scale field variation across the excitonic layers.

### Appendix A.3. Material Parameters

The dispersive dielectric functions of Au and Ag were taken from the experimental optical constants reported by Johnson and Christy and Palik, respectively. The optical response of the J-aggregate molecular layer was described by a Lorentz oscillator model,

(A1) $$\varepsilon_{J}\left(\omega \right)=\varepsilon_{J,\infty }+\frac{f_{J}}{\omega_{J}^{2}-\omega^{2}-i\gamma_{J}\omega },$$

where $\omega_{J}$ is the Frenkel exciton resonance frequency, $\gamma_{J}$ is the damping rate, and $f_{J}$ is the oscillator-strength parameter. In the simulations, the J-aggregate resonance frequency was set to $\omega_{J}=3.1\times 10^{15}\;\mathrm{rad}\;s^{-1}$, and the damping rate was set to $\gamma_{J}=4.3982\times 10^{13}\;\mathrm{rad}\;s^{-1}$.

The monolayer WS_2_ was modeled using the gate-voltage-dependent complex dielectric function developed in this work. This model includes both the linear Stark shift of the WS_2_ exciton resonance and the field-induced reduction of oscillator strength caused by electron–hole wavefunction delocalization. The calculated dielectric function was used as the optical input for the FDTD simulations under different applied gate voltages. All optical parameters used in the simulations are summarized in Table S3. The Au and Ag optical constants were taken from tabulated experimental data, while the J-aggregate and WS_2_ excitonic responses were described using Lorentz oscillator models. The voltage-dependent WS_2_ parameters were obtained from the phenomenological calibration procedure described in the main text.

### Appendix A.4. Spectral Analysis and Coupled-Oscillator Fitting

The resonance wavelengths of the polariton branches were extracted from the simulated extinction or scattering spectra and converted into photon energies using E=hc/λ. The anticrossing dispersions were fitted using the coupled-oscillator Hamiltonian described in the main text. The eigenvalues were used to obtain the polariton energies, while the eigenvectors were used to calculate the Hopfield coefficients.
