# Supplementary material for: Selective Electrical Tuning of Triple-Mode Strong Exciton–Plasmon Coupling in a WS2/J-Aggregates/Au@Ag Heterocavity
Source: Nanomaterials (Basel). 2026 Jun 16;16(12):758. doi: 10.3390/nano16120758 (PMC13306143; doi:10.3390/nano16120758)
Supplement: Supplementary file 1 [file nanomaterials-16-00758-s001.zip › nanomaterials-4347371-supplementary.pdf]

Supporting Information

Selective Electrical Tuning of Triple-Mode Strong Exciton-Plasmon Coupling in a WS2 / J-aggregates / Au@Ag Heterocavity

Yufeng Hu,[†] *Zhiyuan Li,* [†] *Qinglong Peng,*[†] *Chen Xu,* [†] *Yinyin Jiao,* [†] *Lan Jiang,* [†] *and* [†] *Kun Liang*[*] [†]

[†]State Key Laboratory of Information Photonics and Optical Communications, School of Physical Science and Technology, Beijing University of Posts and Telecommunications, 10 Xitucheng Road, Beijing, 100876, China

[*]E-mail: kunliang@bupt.edu.cn;

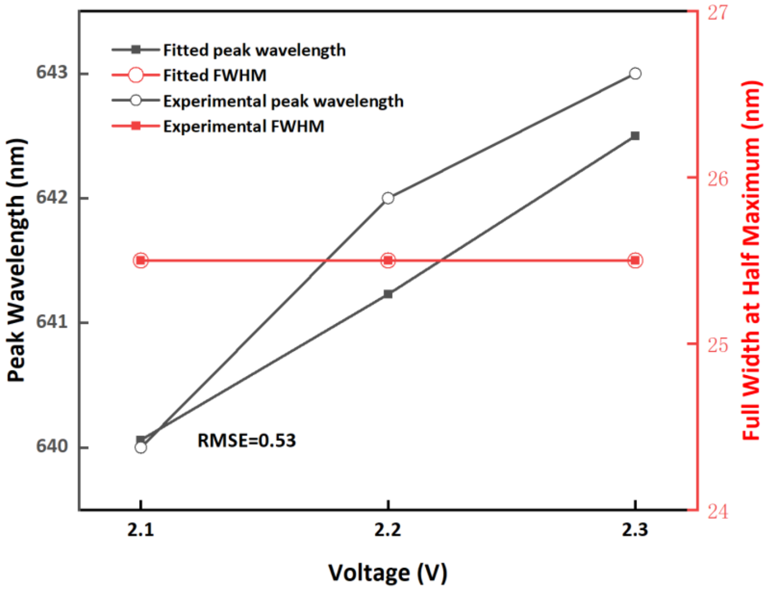

Figure S1. Comparison of the gate-voltage-dependent exciton peak wavelengths and full widths at half maximum (FWHM) extracted from the experimentally reported WS_2 spectra with the corresponding values obtained from the present Lorentz-type dielectric model. The fitted peak wavelengths agree well with the experimental data, with a root-mean-square error of (0.53 nm).

Table S1. The linewidth data of plasmons, J-aggregates, WS_2, and polaritons.

Mode / branch	Peak position	Energy	FWHM	Extraction method
Bare plasmon	592 nm	2.095 eV	124 meV	uncoupled Au@Ag spectrum
Bare J-aggregate exciton	599 nm	2.070 eV	50 meV	uncoupled J-aggregate absorption / Ref [1]
Bare WS_2 exciton	617 nm	2.010 eV	25.6 meV	uncoupled WS_2 absorption /Ref [2]
UPB	577.22 nm	2.148 eV	75.0 meV	coupled spectrum
MPB	606.84nm	2.043 eV	84.6 meV	coupled spectrum
LPB	625.88 nm	1.981 eV	90.4 meV	coupled spectrum

Table S2. Cooperativity analysis.

Coupling channel	g_i	γ_{pl}	γ_i	$C_i=4g_i^2/(\gamma_{pl}*\gamma_i)$	Criterion
Plasmon–J exciton	61.3 meV	124 meV	50 meV	2.42	(C_J>1)
Plasmon–WS_2 exciton	57.8 meV	124 meV	76 meV	1.42	(C_X>1)

Table S3. Material parameters used in the numerical simulations

component	Model	Parameter	Value	Source
Au	Experimental data	$\varepsilon_{Au(\omega)}$	tabulated	Johnson & Christy, Ref [3]
Ag	Experimental data	$\varepsilon_{Ag(\omega)}$	tabulated	Palik(0-2um), Ref [4]
J-aggregate	Lorentz oscillator	$\varepsilon_{\infty,J}$	2.1	Fitted/Ref [1]
J-aggregate	Lorentz oscillator	$\Delta\varepsilon_J$	0.05	Fitted/Ref [1]
J-aggregate	Lorentz oscillator	$\omega_{0,J}$	$3.14 \times 10^{15}\ rad/s$	Fitted/Ref [1]
J-aggregate	Lorentz oscillator	γ_J	$3.798 \times 10^{13} rad/s$	Fitted/Ref [1]
WS_2 (2.0V)	Lorentz-type model	$\varepsilon_{\infty,X}$	4.2	Calibrated/Ref [2]
WS_2 (2.0V)	Lorentz-type model	$\Delta\varepsilon_X(V_0)$	0.31152	Calibrated/Ref [2]
WS_2 (2.0V)	Lorentz-type model	$\omega_{0,X}(V_0)$	$2.9439 \times 10^{15} rad/s$	Calibrated/Ref [2]
WS_2 (2.0V)	Lorentz-type model	$\gamma_X(V_0)$	$5.01358 \times 10^{13} rad/s$	Calibrated/Ref [2]
WS_2	Voltage-dependent model	β	31.1 meV/V	Calibrated/Ref [2]

References

[1] Zengin, G.; Johansson, G.; Johansson, P.; Antosiewicz, T. J.; Käll, M.; Shegai, T. Approaching the Strong Coupling Limit in Single Plasmonic Nanorods Interacting with J-Aggregates. Sci. Rep. 2013, 3, 3074.

[2] Zheng, J.; Krasavin, A. V.; Yang, R.; Wang, Z.; Feng, Y.; Tang, L.; Li, L.; Guo, X.; Dai, D.; Zayats, A.V.; et al. Active control of excitonic strong coupling and electroluminescence in electrically driven plasmonic nanocavities. Sci. Adv. 2025, 11, eadt9808.

[3] Johnson, P. B.; Christy, R. W. Optical Constants of the Noble Metals. J. Phys. Chem. 1972, 76, 437–440.

[4] Palik, E. D., Ed. Handbook of Optical Constants of Solids; Academic Press: Orlando, FL, 1985.
